# Mechanism of action and synergistic effect of *Eugenia uniflora* extract in *Candida* spp.

**DOI:** 10.1371/journal.pone.0303878

**Published:** 2024-08-13

**Authors:** Luanda B. F. C. Souza, Aurélio de Oliveira Bento, Estela M. G. Lourenço, Magda R. A. Ferreira, Wogenes N. Oliveira, Luiz Alberto L. Soares, Euzébio G. Barbosa, Hugo A. O. Rocha, Guilherme Maranhão Chaves

**Affiliations:** 1 Medical and Molecular Mycology Laboratory, Department of Clinical and Toxicological Analyses, Federal University of Rio Grande do Norte, Natal, Brazil; 2 Department of Pharmacy, Federal University of Rio Grande do Norte, Natal, Brazil; 3 Laboratory of Synthesis and Transformation of Organic Molecules, LP4, Institute of Chemistry, Federal University of Mato Grosso do Sul, Campo Grande, MS, Brazil; 4 Department of Pharmaceutical Sciences, Federal University of Pernambuco, Recife, Brazil; 5 Department of Biochemistry, Federal University of Rio Grande do Norte, Natal, Brazil; University of Jeddah, SAUDI ARABIA

## Abstract

The limited arsenal of antifungal drugs have prompted the search for novel molecules with biological activity. This study aimed to characterize the antifungal mechanism of action of *Eugenia uniflora* extract and its synergistic activity with commercially available antifungal drugs on the following *Candida* species: *C*. *albicans*, *C*. *tropicalis*, *C*. *glabrata*, *C*. *parapsilosis* and *C*. *dubliniensis*. *In silico* analysis was performed to predict antifungal activity of the major compounds present in the extract. Minimal inhibitory concentrations (MICs) were determined in the presence of exogenous ergosterol and sorbitol. Yeast cells were grown in the presence of stressors. The loss of membrane integrity was assessed using propidium iodide staining (fluorescence emission). Synergism between the extract and antifungal compounds (in addition to time kill-curves) was determined. Molecular docking revealed possible interactions between myricitrin and acid gallic and enzymes involved in ergosterol and cell wall biosynthesis. *Candida* cells grown in the presence of the extract with addition of exogenous ergosterol and sorbitol showed 2 to 8-fold increased MICs. Strains treated with the extract revealed greater loss of membrane integrity when compared to their Fluconazole counterparts, but this effect was less pronounced than the membrane damage caused by Amphotericin B. The extract also made the strains more susceptible to Congo red and Calcofluor white. A synergistic action of the extract with Fluconazole and Micafungin was observed. The *E*. *uniflora* extract may be a viable option for the treatment of *Candida* infections.

## Introduction

*Candida* spp. are considered yeasts of medical interest, due to the high frequency that they colonize and infect the human host [1]. The genus *Candida* is composed by more than 150 species, while some of them may constitute the normal microbiota of healthy individuals. Under certain circumstances, such as in immunocompromised patients, yeast cells may undergo a transition from commensal to infectious microorganisms, being considered opportunistic pathogens [2].

*Candida* infections may range from superficial lesions of the skin and mucous membranes to life-threatening disseminated systemic diseases. *Candida albicans* is the main isolated species from different body sites [3]. However, it is important to note that non-*Candida albicans Candida* (NCAC) species such as *Candida glabrata*, *Candida tropicalis*, *Candida parapsilosis* and *Candida krusei* have also been frequently isolated as etiological agents of several clinical forms of candidiasis. More recently, a newly described *Candida* species named *Candida auris*, initially isolated from an ear infection has been object of concern due to its multidrug resistance to antifungal agents of different therapeutic classes [4–7].

The arsenal of antifungal agents commercially available is limited when compared to the wide variety of antibacterial drugs. Furthermore, there is a limitation in the development of new active antifungal agents that do not cause toxic effects to the human host, because fungal cells are also eukaryotic [8]. Briefly, antifungal drugs may be mainly classified into different categories, according to their mechanism of action and cellular target, as follows: 1) Polyenes (bind to ergosterol in fungal cell membranes, causing leakage of intracellular ions). 2) Azole derivatives (interact with 14-demethylase, leading to the accumulation of toxic sterol). 3) Morpholines (inhibit the Δ-14-sterol-reductase, an enzyme of ergosterol biosynthesis pathway). 4) Echinocandins (inhibition of the enzyme 1,3-β-glucan synthase, a critical cell wall component) and 5) Antimetabolites agents (target fungal DNA replication and RNA synthesis, including 5-fluorocytosine) [8, 9].

Due to the limitation of antifungals available on the market, their adverse effect and the emergence of antifungal resistance, there is a constant need to search for new molecules that may act on alternative fungal cell targets, causing less toxicity to mammalian hosts [10–12]. In this context, the investigation of herbal medicines and natural products have become increasingly high worldwide and may be a valuable source of new antimicrobial agents [13, 14].

*Eugenia uniflora* is a plant native of South America, belonging to the Myrtaceae family, popularly known in Brazil as "pitangueira". This plant has several biological activities described, such as antidiabetic, antirheumatic, antidiarrheal, anti-inflammatory, hepatoprotective activity, anti-trypanosomal, antifungal, antibacterial and antioxidant. In addition, there are also reports of its use in the treatment of gastrointestinal diseases [15–19].

Recently, the extract obtained from the leaves of *E*. *uniflora* had its anti-*Candida* activity described in the literature [20, 21]. Other studies have reported the action of plant products derived from *E*. *uniflora* on the expression of virulence factors by *Candida* spp. *in vitro* and *in vivo*, such as: morphogenesis and secretion of hydrolytic enzymes [15], adhesion to humman buccal epitelial cells [17] and the impairment of biofilm formation [17, 21].

In the process of discovering novel antifungal compounds, it is necessary to understand their direct mechanism of action on fungal cells [22, 23]. For the achievement of this purpose, *in vitro*, *in vivo* and *in silico* techniques have been used to elucidate such mechanisms [8, 24]. Furthermore, it is worth mentioning that naturally occurring active compounds may frequently be more useful as chemotherapeutic agents when used in combination with other substances, rather than as a monotherapy. Therefore, some plant derivatives have been investigated for their possible synergism with azoles, polyenes or echinocandins currently used to treat *Candida* infections [25].

The global incidence of invasive *Candida* infections has increased in the last decade and is dependent upon geographical location and patient population. Recent global estimates have suggested that approximately 700,000 cases of invasive candidiasis occur annually [26]. Therefore, the discovery of novel natural products would benefit immunocompromised individuals highly colonized by *Candida* spp., who may be at risk of further developing candidiasis.

Thus, this study aimed to elucidate the antifungal mechanism of action of the *E*. *uniflora* extract (EuE) on *Candida* spp. cells. Besides, we also evaluated a possible synergistic activity when this plant derivative was tested in combination with commercially available antifungal drugs.

## Material and methods

### Ethics statement

Of note, it was not necessary a research ethics committee approval for this study, once that these strains were not collected from patients, but provided by culture collections. Therefore, no permits were required for the described study, which complied with all relevant regulations.

### Vegetal material and *Eugenia uniflora* extract obtaining

The leaves of *E*. *uniflora* were collected in the state of Rio Grande do Norte, Northeast Brazil, in July 2009. The plant material was identified in the Herbarium of the Federal University of Rio Grande do Norte (Department of Botany, Zoology and Ecology, Biosciences Centre) and one vouch specimen was deposited under the number 11763. The extract was obtained from dried leaves, which were subjected to a milling process. Then, 10 g of the powder obtained from the leaves were placed in a turbo-extractor with 100 ml of acetone: water (7: 3, v/v) four times, during five minutes and the obtained extract was filtered and concentrated in an evaporator (Laborota 4000 Heidolph) at 40°C, 150 rpm. The EuE was lyophilized and subsequently stored at 4°C, as previously described [17].

### *Eugenia uniflora* extract chemical characterization

The EuE was analyzed on an HPLC equipped with Diode Array Detector (DAD), binary pump, degasser, and autosampler (Ultimate 3000 Thermo Fisher Scientific®). The Chromeleon software (Dionex®) was used for data acquisition and processing. Chromatographic separation was performed on a C18 column (250 mm x 4.6 mm, 5 μm; Supelco®) protected by a security guard of the same composition (Phenomenex®). The mobile phase consisted of ultrapure water (A) and methanol (B), both acidified with 0.05% (v/v) trifluoroacetic acid (Vetec®, Brazil), and was used a gradient condition as following: 0–10 min, 10–25% B; 10–15 min, 25–40% B; 15–25 min, 40–70% B; 25–30 min, 75% B and 30–31 min, 75–10%, at a flow rate of 0.8 mL/min. The chromatographic separations were performed at 24 ± 2°C and wavelength of 270 and 350 nm were used to detect gallic acid (96% purity, Sigma®), and myricitrin (99% purity, Sigma®), respectively, according to the absorption maximum measured by DAD [27].

### Strains and culture conditions

We evaluated four *Candida* spp. strains belonging to the American Type Culture Collection (ATCC), as follows: *C*. *albicans* ATCC 90028, *C*. *tropicalis* ATCC 13803, *C*. *glabrata* ATCC 2001, *C*. *parapsilosis* ATCC 22019 and a single strain of *Candida dubliniensis*, originally obtained from the Westerdijk Fungal Biodiversity Institute (CBS 7987). The isolates were stored at -80°C in YPD containing 20% ​​glycerol. The strains were initially thawed on ice and 100 μL of cells suspensions was transferred to conical tubes containing 5 ml of YPD liquid medium (dextrose 20 g/L, peptone 20 g/L, yeast extract 10 g/L) and further incubated in a rotary shaker (TE-420, Tecnal® Piracicaba, Brazil) at 35°C, 200rpm, for 48h for reactivation and viability verification. After the incubation period, 100 μL of fungal cell suspensions were inoculated onto the surface of Sabouraud Dextrose Agar (SDA; Oxoid, UK) using a Drigalsky loop. The plates were incubated at 37°C for 48h. Yeast colonies were streaked out on a chromogenic medium (CHROMagar Microbiology, Paris, France) to check for purity [28].

### *In silico* analyzes and molecular docking simulations

The 3D structures of the main compounds found in the leaf extract of EuE (gallic acid and myricitrin) were drawn using the MarvinSketch 16.9.5 software (ChemAxon Ltd.). The geometric optimization was performed in the MOPAC2016 software using the semi-empirical method PM7. The structures were subjected to molecular docking simulations. The enzymes 14-α-demethylase (PDB ID: 3JUV), Δ-14-sterol-reductase (PDB ID: 4QUV), β 1,3 D-glucan synthase (PDB ID: 2JUV) and thymidylate synthase (PDB ID: 3QJY) were the molecular targets investigated in our study. All crystallography proteins were obtained from the Protein Data Bank. Molecular docking simulations were performed using AutoDockVINA software, with a grid box large enough to contain the target’s active site [24].

### Antifungal susceptibility profile of *Candida* spp. to commercial antifungal drugs and *Eugenia uniflora* extract

Solutions of fluconazole (FLU), amphotericin B (AMB), micafungin (MCF) and EuE were prepared in accordance with guidelines M27-A3 from the Clinical and Laboratory Standards Institute (CLSI), by being diluted in RPMI 1640 (Roswell Park Memorial Institute; Angus buffers and Biochemical, Niagara Falls, NY, USA) buffered 3-(N-morpholino) propanesulfonic acid (MOPS) to pH 7.0. Antifungal drugs tested were diluted serially in 10 different concentrations, namely: FLU (Pfizer Incorporated, New York, NY, USA) 0.125–64 μg/mL; AMB (Sigma Chemical Corporation, St. Louis, MO, USA) 0.013–16 μg/mL; MCF (Merck, Rahway, NJ, USA) 0.015–8 μg/mL and EuE (10,000–78.1 μg/mL). The inocula of all strains tested were obtained from 48 h cultivation in Sabouraud broth at 30°C and an initial cellular suspension in saline solution equivalent to the 0.5 MacFarland standard, determined spectrophotometrically at 530 nm. Then, two serial dilutions were made, the first in saline solution (1:100) and the second in RPMI (1:20), in order to obtain a final concentration of 10^3^ cells/mL. Aliquots of 100 μL of the final inoculum solution were dispensed in microtiter plates of 96 wells containing 100 μL of various concentrations of the tested drugs. Finally, the plates were incubated at 37°C and test reading taken after 48 h incubation. All strains were tested in duplicate. MIC was defined for FLU, MCF and EuE to the lowest drug concentration which showed approximately 50% reduction in turbidity as compared to the positive control well. For AMB, the MIC was defined as the lowest concentration able to inhibit any growth visually perceptible [29].

### Interaction of *Eugenia uniflora* extract with fungal membrane ergosterol (ergosterol assay)

In order to investigate whether the EuE binds to fungal membrane sterols, microdilution tests for minimal inhibitory concentrations (MICs) determinations were performed with the addition of an exogenous source of ergosterol. The assay was performed according to the protocol described in the Clinical and Laboratory Standards Institute (CLSI) document [29]. The ergosterol powder (Sigma-Aldrich®) was solubilized in dimethylsulfoxide (DMSO, Sigma-Aldrich®) and Tween 80 (Sigma-Aldrich®) in a ratio of 4:1 (v/v). The emulsion was heated up to 55°C and further vigorously homogenized, prepared during the moment of experimentation. The solution was added to the RPMI-1640 culture medium (Roswell Park Memorial Institute, Vitrocell), and the mixture was filtered through a 0.2 μm filter (Merck Millipore). One hundred microliters of the resulting solution was distributed to 96-wells polystyrene plates and another 100 μL of standardized *Candida* cells suspension (10^3^ cells/mL) was added to the wells (as previously described above). The final exogenous ergosterol concentration in each well was 100 μg/mL and the EuE was tested in the concentration range from 10,000 to 78.1 μg /mL. The plates were incubated for 24–48 h at 37°C. AMB was used as a control for exogenous ergosterol binding. The tests were performed in duplicate, and the MICs were considered as the lowest concentration of the substance that inhibited 50% of visible growth in the presence of the EuE with the addition or in the absence of exogenous ergosterol [30].

### Effect of *Eugenia uniflora* extract on fungal cell wall (sorbitol assay)

The effect of the EuE on the cell wall of *Candida* spp. strains was analyzed with the addition of sorbitol as an osmoprotectant. Sorbitol at the final concentration of 0.8 M (D-sorbitol anidro, Sigma Aldrich St. Louis, MO, EUA) was added to 96-wells polystyrene plates containing RPMI-1640 culture medium (100 μL of total volume) together with another 100 μL of standardized *Candida* cells suspension (10^3^ cells/mL) and the EuE tested in the concentration range from 10,000 to 78.1 μg /mL. The plates were incubated for 24–48 h at 37°C. MCF was used as a positive control as it targets *Candida* spp. cell wall (8 μg/mL). The tests were performed in duplicate, and the MICs were considered as the lowest concentration of the substance that inhibited 50% of visible growth in the presence of the EuE with the addition or in the absence of exogenous sorbitol [30].

### Interactions of *Eugenia uniflora* extract with fungal plasma membrane by flow cytometry analysis

*Candida* spp. strains were cultivated in YPD broth medium, in the presence (test) and absence (control) of 1000 μg/mL of *E*. *uniflora* extract for 48 h at 30°C in a rotary shaker (Tecnal, TE-420) at 200 rpm. After the incubation period, the cells were washed with 1 mL of PBS and centrifuged (Excelsa®4, MOD 280R) for 6 minutes at 2500 rpm. Subsequently, washed fungal cells were fixed in 1mL of methanol (P.A, Vetec) for 45 min and further washed with 1mL of PBS under the same centrifugation conditions. Finally, fungal cells were stained with 5 μL of propidium iodide solution (PI, 100 μL/mL, Invitrogen) and the fluorescence intensity assessed by flow cytometry (FACSCanto II, BD Biosciences, OR, USA) with emission in 630–622 nm for PI with 30,000 events acquired. Untreated cells of *C*. *albicans* ATCC 90028 were used as a negative control and cells treated with FLU (2 μg/mL) and AMB (1 μg/mL) were used for cell membrane damage investigations, determined as the percentage of fluorescence emission [31]. Triton-X (0.01%) was used as a positive control for membrane permeabilization to normalize the results [32]. The fluorescence intensity values of the negative controls were compared to the test samples and statistical analyzes were performed in order to verify differences in the intensity of fluorescence emitted between control and test experiments.

### Fungal cell wall stressors assay

*Candida* strains were cultured in YPD broth, in the presence (test) and absence (control) of 1000 μg/mL of the EuE for 48h at 30°C on a rotary shaker (Tecnal, TE-420) at 200 rpm. Inoculum standardization was performed in a spectrophotometer (Biochrom Libra S32) at a wavelength of 600 nm, where the optical density (O.D.) of the cell suspensions was adjusted to 0.8. Then, ten-fold serial dilutions (10^−1^ to 10^−5^) were prepared in phosphate buffered saline (PBS) and 5 μL aliquots were inoculated onto the surface of YPD agar plates containing the following cell wall stressors: Calcofluor white (38 μg/mL; Sigma Aldrich), Congo red (350 μg/mL; Sigma Aldrich) and sodium dodecyl sulfate (SDS; 18 μg/mL; Sigma Aldrich). The plates were incubated for 48 h at 37°C. The assay was performed in duplicate and test and control plates were visually compared for the observation of colony forming units (CFU) growth inhibition after *Candida* cells incubation in the presence and absence of the EuE and further inoculation on the plates containing each cell wall stressor [33].

### Synergistic effect of *Eugenia uniflora* extract combined with synthetic antifungal drugs and gallic acid

Possible synergistic effects of *E*. *uniflora* extract with FLU, AMB, MFC and the reference substance of gallic acid (PA, Vetec, 10,000 to 78.1 μg /mL) were tested in duplicate, by using the combination method known as “Checkerboard”. The assay was performed in 96-wells polystyrene plates. Dilutions of antifungal drugs and gallic acid were prepared to obtain a concentration four times greater than the final concentration within each well, and then two-fold serial dilutions were carried out in the microplate to obtain the concentration ranges previously mentioned. The EuE dilutions were prepared in order to obtain a concentration range from 10,000 to 78.1 μg /mL mg/mL. For two-dimensional microplate preparation (i.e., antifungal/gallic acid versus *E*. *uniflora* extract), 50 μl of each concentration of antifungal drug/gallic acid was added to columns 1–10. Subsequently, 50 μl of the EuE dilutions were added to rows A to G. Row H and column 11 contained only either the antifungal drug or gallic acid and the EuE, respectively. Finally, 100 μl of the *Candida* cells suspension was added to each well, with the exception of the sterility control. Half of column 12 was used to the control of growth (culture medium containing yeast cells suspension) and the other half to sterility control (containing only the culture medium). The microtiter plates were incubated at 37°C and visual readings were taken 24–48 h after incubation. The fractional inhibitory concentration index (FICI) was used to interpret the results. The FICI was calculated using the following equation: FICI = FIC A + FIC B, where ’A’ represents the test product and ’B’ the standard antifungal drug or gallic acid. The FICA is calculated by the ratio of combined MICA to the MICA alone, while the FICB is calculated by the ratio of combined MICB to the MICB alone. The interaction was defined as synergistic, indifferent and antagonistic if the FICIs were ≤ 0.5, > 0.5 to ≤ 4.0 and > 4, respectively [34].

### Time-kill curve of *Candida* spp. after treatment with the *Eugenia uniflora* extract combined with antifungal drugs

*Candida* cells were initially cultured on Sabouraud Dextrose Agar (SDA, Difco) for 24–48 h. Subsequently, a cell suspension was prepared in sterile saline 0.85% solution, to standardize the inoculum to 1.0 x 10^5^ CFU/mL. After the preparation of fungal cell suspensions, 100 μL aliquots were added to conical tubes containing 5 mL of YPD medium, as follows: 1) Control experiment (YPD culture medium only). 2) EuE alone (1000 μg/ mL). 3) EuE (1000μg/mL) combined with FLU (8μg/mL). 4) FLU alone (8 μg/mL). 5) EuE (1000 μg/mL) combined with MCF (1μg/mL) and 6) MCF alone (1μg /mL). The tubes were incubated with shaking at 30°C, 200 rpm and aliquots of 100 μL in 10-fold serial dilutions cells were resupended in sterile saline solution at different time points (0, 8, 16, 24, 36 and 48 h). Subsequently, the whole volume of each dilution was streaked out on the surface of SDA plates with the aid of a Drigalsky loop. Colony counts were determined after plates incubation at 30°C for 48 h. Fungicidal activity was defined as a ≥ 3 log 10 reduction of the initial inoculum. Synergism and antagonism were defined as the decrease or increase of ≥ 2 log10 CFU/mL counts respectively, produced by the combined preparation, compared to that of the active agent alone [34]. The assays were performed in duplicate.

### Statistical analysis

The Shapiro-Wilk test was performed to assess the normal distribution of continuous variables. Variables with parametric distribution were analyzed with Student’s T test or One-way ANOVA, followed by Tukey’s post-test and Pearson’s correlation. Variables considered non-parametric were analyzed using the Mann-Whitney or Kruskall-Wallis tests, followed by Dunn’s post-test and by the Spearman correlation. All the statistical analyses were performed with the GRAPHPAD PRISM, version 6.0 software (GraphPad Software, Inc., San Diego, CA, USA). For all statistical analyzes performed, the results whose descriptive levels (*P* values) were lower than 0.05, were considered statistically significant.

## Results

In a previous study carried out by our group, the chromatographic profiling of the EuE revealed two major compounds, namely: gallic acid and myricitrin [17]. Based on these findings, here we investigated the possible mechanisms of action of the anti-*Candida* activity of *E*. *uniflora* on fungal cells, specifically of the main majoritary compounds found.

### *In silico* analysis of the majoritary compounds antifungal action of the *Eugenia uniflora* extract

Based on the well-known mechanism of action of commercially available antifungal drugs, the following enzymes were chosen as putative molecular targets of each majoritary compound on *Candida* cells: 14-α-demethylase (PDB ID: 3JUV), Δ-14- sterol reductase (PDB ID: 4QUV), 1,3-β-glucan synthase (PDB ID: 2JUV) and thymidylate synthase (PDB ID: 3QJY).

**Fig 1:**
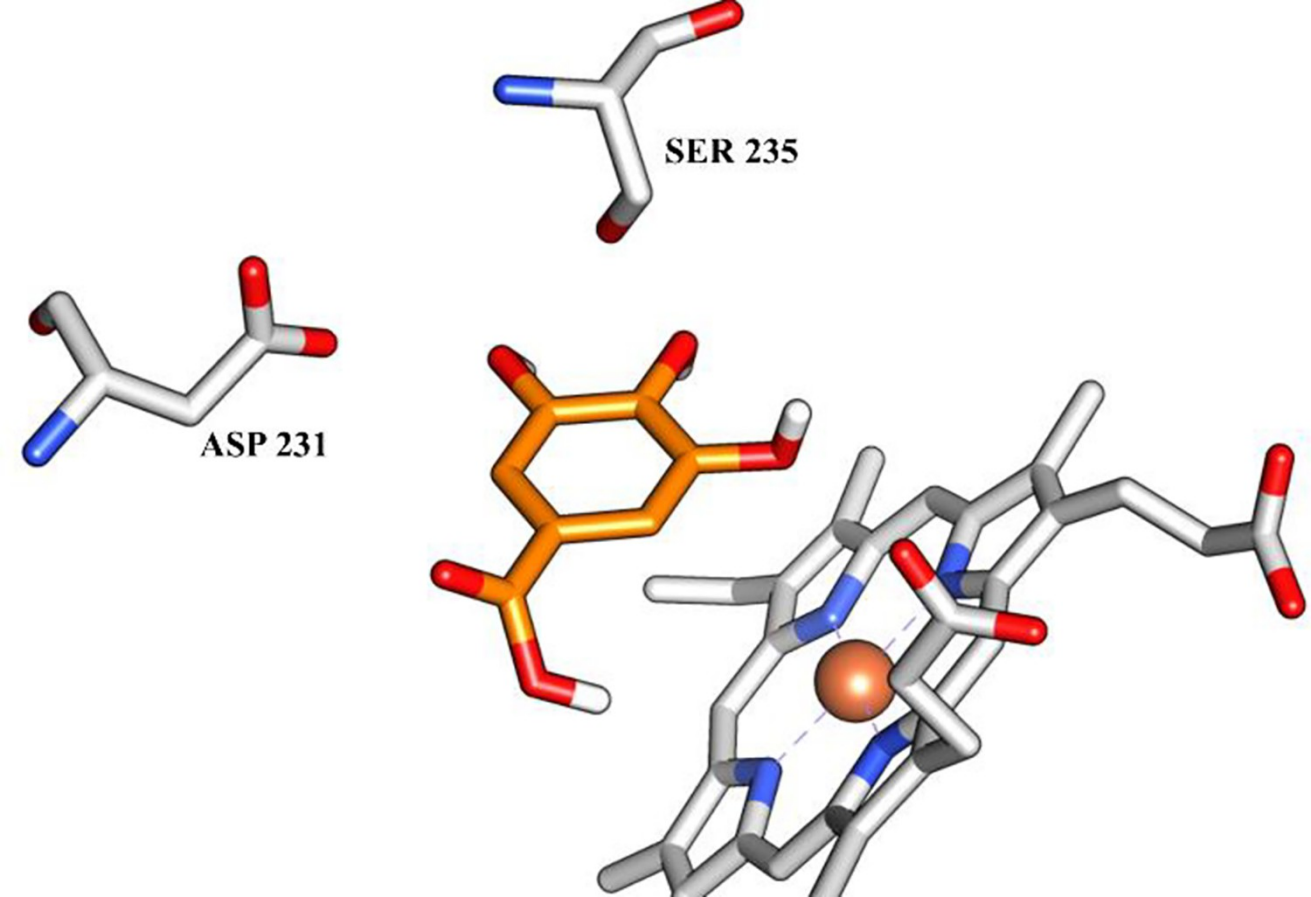
Intermolecular interactions between gallic acid (orange) and the amino acid residues of Δ-14-sterol-reductase (PDB ID: 4QUV).

Differences in the molecular framework of gallic acid and myricitrin result in their distinct ability to interact intermolecularly with targets. Gallic acid showed a potential interaction only at the active site of Δ-14-sterol-reductase. Even possessing low molecular weight, the gallic acid showed hydrogen bonds with SER 235 and ASP 231 and possible interactions with the heme group (Fig 1).

**Fig 2:**
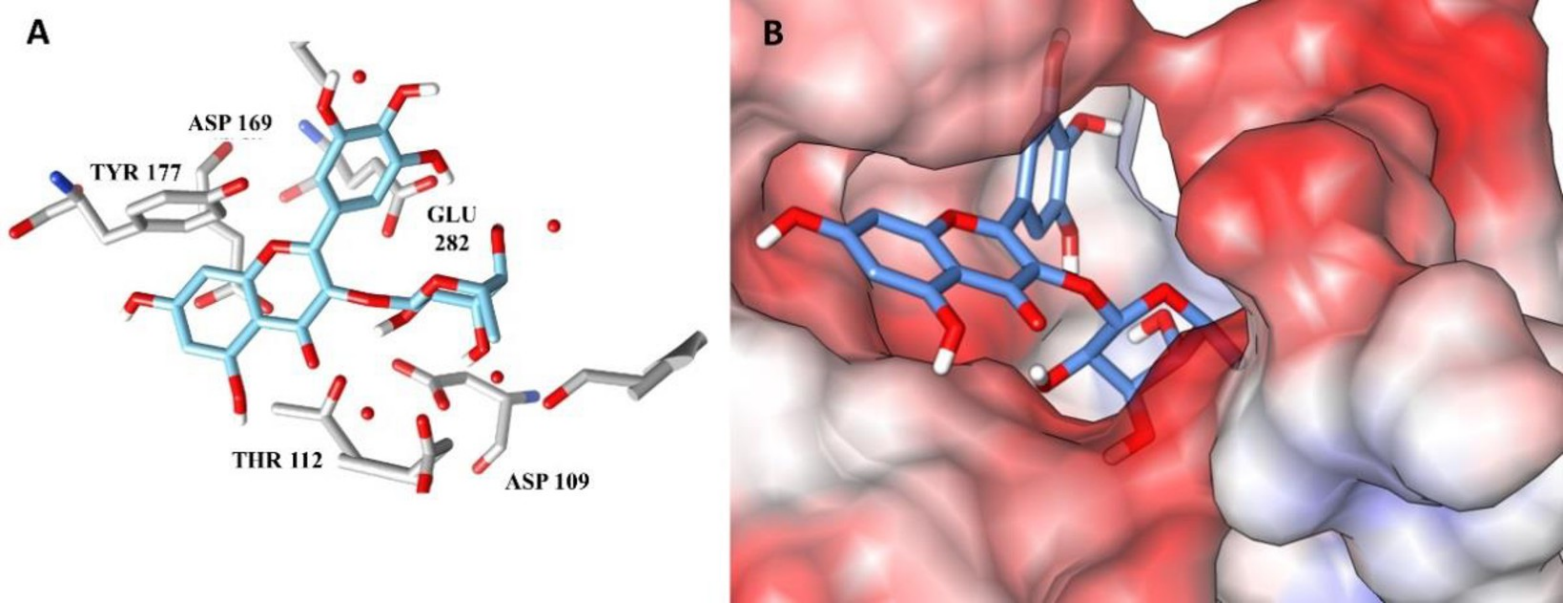
(A) Intermolecular interactions and (B) Binding mode position of myricitrin (blue) with the 1,3-β-glucan-synthase (PDB ID: 2JUV).

**Fig 3:**
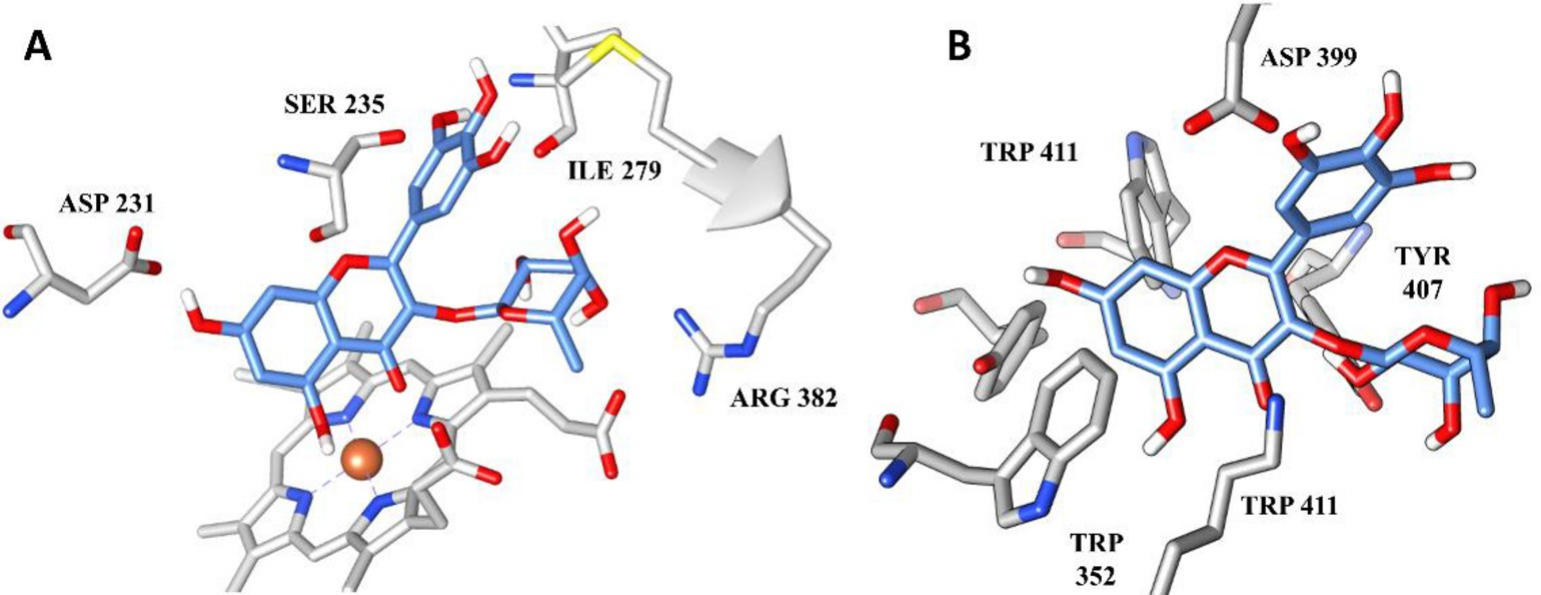
(A) Intermolecular interactions between myricitrin (blue) with the amino acid residues of Δ-14-sterol-reductase (PDB ID: 4QUV) and (B) 14-α-demethylase (PDB ID: 3JUV).

In contrast, myricitrin had a likely interaction with three out of the four molecular targets tested. It is important to emphasize that the presence of several hydroxyl groups in its structure may favor the formation of hydrogen bonds between targets and polar amino acid residues. Since a strong intermolecular interaction is considered, the compound forms a stable occupation at the target binding site, explaining its multitarget characteristic. Hydrogen bonds were identified in large numbers between myricitrin and the amino acid residues of 1,3-β-glucan synthase (GLU 282, ASP 169, TYR 112 and ASP 109) and consequently the compound occupied the polar pocket of the target (Fig 2A and 2B, respectively). Furthermore, myricitrin also showed possible interactions with Δ-14-sterol reductase (Fig 3A) and 14-α-demethylase (Fig 3B) mainly through hydrogen bonds with the amino acid residues SER, ASP, ARG and TRP. No compound showed interaction with the enzyme thymidylate synthase (PDB ID: 3QJY).

### Exogenous ergosterol binding assay on fungal cell wall in the presence of the *Eugenia uniflora* extract

In order to verify whether the antifungal action of the EuE extract is related to its direct binding on *Candida* cells plasma membrane, exogenous ergosterol was added to the broth microdilution antifungal susceptibility test with yeast cells grown in the presence of the extract. Exogenous source of ergosterol may increase the MIC value for compounds that target this sterol in the cell membrane, once it will compete directly with the ergosterol present in fungal membranes [30]. AMB (a polyene drug) was used as an ergosterol binding control due to its known mechanism of action by forming complexes with ergosterol located in fungal membranes leading to an hydro-electrolytic imbalance and consequently potassium leakage, causing lysis and cell death [35]. In the present study, the MIC values of the EuE against *Candida* spp. ranged from 312.5 μg/mL to 625 μg/mL, while all strains showed an MIC equal to 0.03 μg/mL against AMB (in the absence of addition of exogenous ergosterol). When *Candida* cells were grown in the presence of exogenous ergosterol in the same conditions previously mentioned, the MICs of the EuE doubled for most of the strains tested except for *C*. *tropicalis* strain ATCC 13803, which remained unaltered. Nevertheless, when cells were grown in the presence of AMB and exogenous ergosterol was added, the MICs increased for all the strains tested, up to 16-fold higher concentration (Table 1).

**Table 1:** Minimal inhibitory concentrations of *Eugenia uniflora* extract against *Candida* spp. in the presence of exogenous ergosterol determined by the broth microdilution method.

	Minimal Inhibitory Concentration			
Strains	*E*. *uniflora* extract	*E*. *uniflora* extract + Ergosterol	Amphotericin B	Amphotericin B + Ergosterol
***Candida albicans* ATCC 90028**	312.5 μg/mL	625 μg/mL	0.03 μg/mL	0.25 μg/mL
***Candida dubliniensis* CBS 7987**	312.5 μg/mL	625 μg/mL	0.03 μg/mL	0.25 μg/mL
***Candida tropicalis* ATCC 13803**	625 μg/mL	625 μg/mL	0.03 μg/mL	0.50 μg/mL
***Candida parapsilosis* ATCC 22019**	312.5 μg/mL	625 μg/mL	0.03 μg/mL	0.25 μg/mL
***Candida glabrata* ATCC 2001**	625 μg/mL	1,250 μg/mL	0.03 μg/mL	0.50 μg/mL

### Sorbitol osmoprotection assay on fungal cell wall in the presence of the *Eugenia uniflora* extract

In the present study, the MIC values of the EuE were analyzed when *Candida* spp. cells were grown in the presence and absence of sorbitol, at a concentration of 0.8 M. Sorbitol is considered an osmotic protector of fungal cells once it is an agent that stabilizes the osmotic pressure of the cell. Thus the MIC values of strong cell wall inhibitors are believed to be increased in their presence [30]. Therefore, it was used to verify a possible mechanism of action of the extract tested directly on fungal cell wall. MCF was chosen as a control, due to its known impairment of proper 1,3-β-glucan synthase function, preventing the proper assembly of the fungal cell wall components [36]. As previously mentioned, the MIC values of the EuE against *Candida* spp. ranged from 312.5 μg/mL to 625 μg/mL, whereas all strains showed an MIC equal to 0.016 μg/mL against Micafungin. Nevertheless, most of the strains showed increased MICs when sorbitol was added to the experimental conditions (2 to 8-fold increase) except *C*. *glabrata* (ATCC2001). All *C*. *Candida* spp. strains had 8-fold increased MICs (0.125 μg/mL), when Sorbitol was added to the assay containing MCF (but no EuE; Table 2).

**Table 2:** Minimal inhibitory concentrations of *E*. *uniflora* extract against *Candida* spp. in the presence of sorbitol determined by the broth microdilution method.

	Minimal Inhibitory Concentration			
Strains	*E*. *uniflora* extract	*E*. *uniflora* extract + Sorbitol	Micafungin	Micafungin + Sorbitol
***Candida albicans* ATCC 90028**	312.5 μg/mL	2,500 μg/mL	0.016 μg/mL	0.125 μg/mL
***Candida dubliniensis* CBS 7987**	312.5 μg/mL	625 μg/mL	0.016 μg/mL	0.125 μg/mL
***Candida tropicalis* ATCC 13803**	625 μg/mL	2,500 μg/mL	0.016 μg/mL	0.125 μg/mL
***Candida parapsilosis* ATCC 22019**	312.5 μg/mL	1,250 μg/mL	0.016 μg/mL	0.125 μg/mL
***Candida glabrata* ATCC 2001**	625 μg/mL	625 μg/mL	0.016 μg/mL	0.125 μg/mL

### Interactions of the *E*. *uniflora* extract with fungal plasma membrane integrity determined by flow cytometry

**Fig 4:**
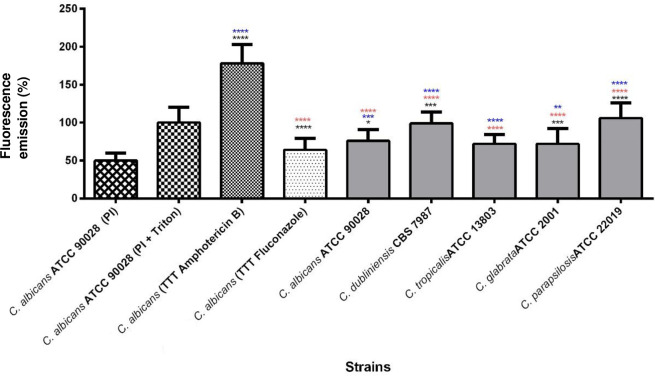
Effect of *E*. *uniflora* extract (1000 μg/mL; 5 right bars), Fluconazole and Amphotericin B on the integrity of *Candida* spp. plasma membrane determined by the penetration of PI after treatment of fungal cells for 48 h, 37°C, at 200 rpm. The bars represent mean ± standard deviation of the percentage of fluorescence emission (of cells with loss of membrane integrity). The assay was performed in triplicate. * *P* <0.05 compared to the control of (*untreated cells); (*Amphotericin B-treated cells) and (*Fluconazole-treated cells) using the Kruskall-Wallis test, followed by Dunn’s post-test.

To further investigate whether the EuE causes damage to the *Candida* spp. plasma membrane, we have used the cytofluorimetric marker PI. *Candida* cells were treated with either the EuE (1,000 μg/mL), or with the antifungal drugs AMB (1 μg/mL) and FLU (2 μg/mL), which served as references to compare the intensity of fluorescence emitted by fungal cells grown in the presence of the extract. It is important to mention that fluorescence emission is related to the loss of *Candida* spp. membrane integrity, because PI is unable to penetrate cells with intact plasma membrane. It is a small fluorescent molecule that binds to DNA, but cannot passively traverse into cells that possess an intact plasma membrane [37]. All *Candida* spp. strains tested were able to emit higher fluorescence after treatment with the EuE when compared to the untreated (previously grown in the absence of the EuE) and PI-labeled control counterparts (*C*. *albicans* ATCC90028). In addition, the positive control Triton-X showed the double of fluorescence emission of *C*. *albicans* ATCC90028 without treatment with the EuE extract. Fungal cells treated with the EuE comparisons with synthetic antifungal drugs (AMB or FLU) revealed greater fluorescence emission than when they were grown in the presence of FLU, but lower fluorescence emission in the presence of AMB (Fig 4).

### Evaluation of fungal cell stressors in the presence of the *Eugenia uniflora* extract

**Fig 5:**
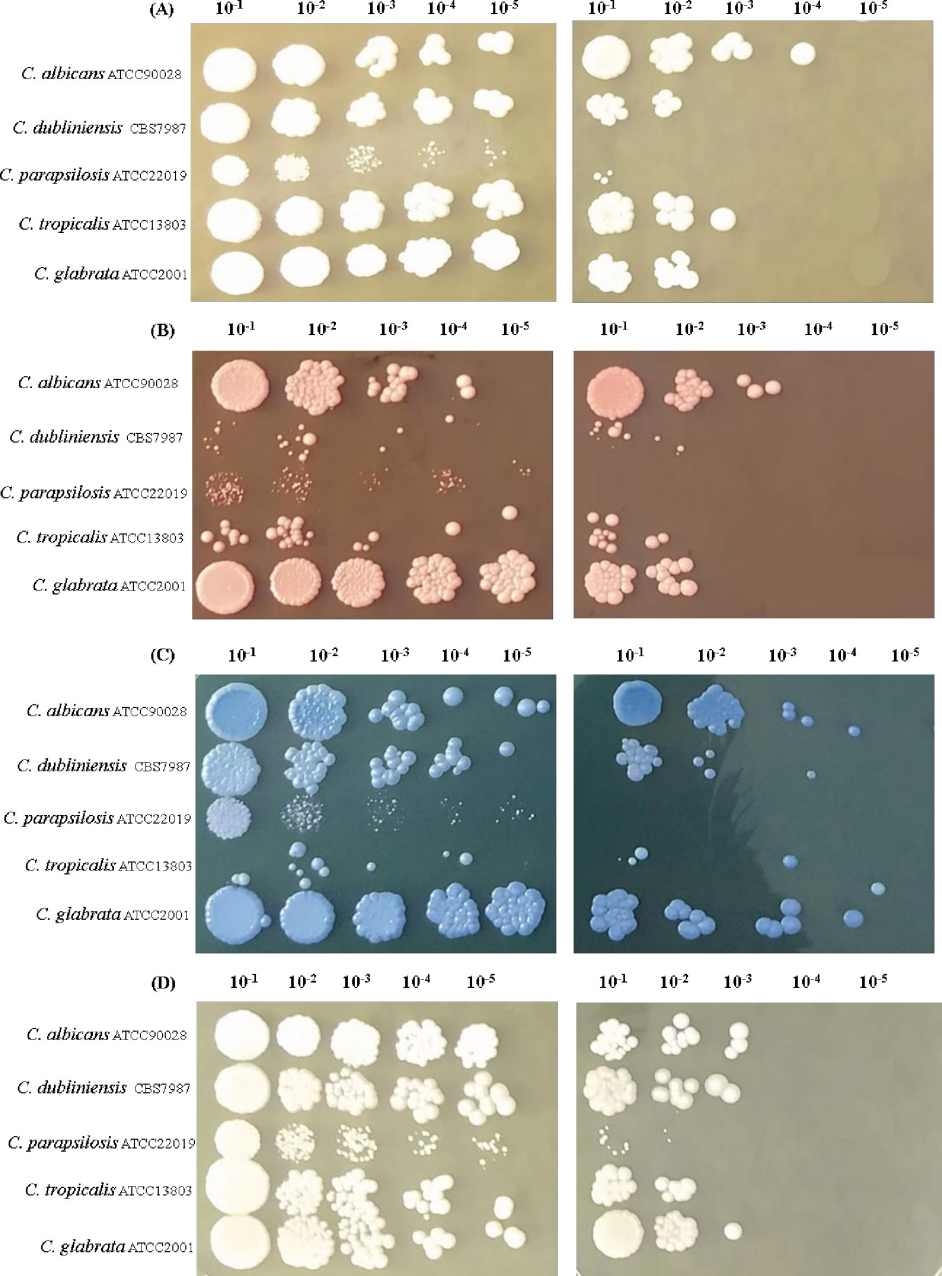
Effect of *Eugenia uniflora* on the ability of *Candida* spp strains growth in the presence of cell wall stressors A) YPD alone. B) YPD containing Congo red (38 μg/mL). C) YPD containing Calcofluor white (350 μg/ml). D) YPD containing SDS (18 μg/ml). The plates were incubated at 30° C for 48 h. The left columns represent the results of strains previously grown in the absence of the extract while in the right columns cells were pre-grown in YPD broth containing the *E*. *uniflora* extract (1000 μg/mL) for 24 h, 37°C, 200 rpm.

The next step was to continue to evaluate plasma membrane and cell wall likely damage caused by the extract of the EuE to *Candida* spp. strains tested in the present study. For these purposes, yeasts were initially grown in the presence and absence of the EuE and further 10-fold serially diluted in saline solution. Five microliters of cellular suspensions was spotted on the surface of YPD agar (control experiment), as well as YPD agar containing cell stressors. As expected, *Candida* spp. strains previously grown in the presence of the extract (test experiment) showed reduced number of CFU counts when compared to their negative counterparts (where no EuE was added to the YPD broth pre-inoculum), after YPD agar plates (alone) incubation. It is worth mentioning that *C*. *parapsilosis* ATCC 22019 barely grew in the test experiment. However, this strain also had poorer growth in the control experiment (Fig 5A).

Not surprisingly, even when the strains were previously grown in the absence of the extract (YPD broth pre-inoculum), there was a reduction in CFU counts after incubation of the spots on YPD Agar plates containing Congo red and Calcofluor white (Fig 5B and 5C). This reduction was less pronounced in *C*. *glabrata* ATCC 2001. Nevertheless, growth inhibition was more pronounced when the strains were previously grown in the presence of the extract and further spotted on the stressors containing media. For instance, Congo red plates showed that, except for C. *albicans* ATCC 90028, all the strains only grew in the 10^−2^ dilution, whereas *C*. *parapsilosis* ATCC 22019 showed no growth at all. The same trend of growth inhibition was observed for Calcofluor white plates, although this inhibition was less pronounced (Fig 5B and 5C).

Growth inhibition was also observed in the presence of SDS (sodium dodecyl sulfate). However, when the pre-inoculum cells were grown in the absence of the EuE plates, they were only slightly affected. On the other hand, test experiments revealed that most of the strains were able to grow only up to the 10^−3^ dilution, whereas *C*. *parapsilosis* ATCC 22019 and *C*. *tropicalis* ATCC 13803 CFU colonies could only be observed for the 10^−2^ dilution (Fig 5D).

### Analysis of a possible synergistic effect between the *Eugenia uniflora* extract and synthetic antifungal drugs in *Candida* spp.

We further investigated if there is a synergistic action between the EuE extract and the following antifungal drugs: FLU, AMB and MCF, as well as to the substance gallic acid, by using the “checkerboard” methodology. The FICI (Fractional Inhibitory Concentration Index) was determined, where the interaction is defined as synergistic, indifferent or antagonistic if the FICI is ≤ 0.5, > 0.5 to ≤ 4.0 and > 4, respectively.

When *Candida* spp. cells were grown in the presence of the EuE and FLU, a 4x synergistic effect was found for most of the strains, whereas no interaction was found between the extract and the drug for *C*. *dubliniensis* CBS7987 and *C*. *parapsilosis* ATCC 22019 (Table 3). A similar trend of synergistic effect was found for MCF and the extract, but the synergistic effect was also observed in *C*. *parapsilosis* ATCC 22019 (Table 4). However, no interactions were found when the EuE and Amphotericin B were tested in combination (Table 5). Despite gallic acid MICs were lower than those found for the EuE for most of the strains, there was no synergistic effect or interaction between the extract and this substance also (Table 6).

**Table 3:** Synergistic activity of the *E*. *uniflora* extract with Fluconazole on *Candida* spp. MIC values of *E*. *uniflora* extract and Fluconazole obtained in the test, calculation of the FICI and the effect resulting from the interaction of the substances. Values obtained using the checkerboard technique after incubation at 37°C for 48 h.

		Minimal Inhibitory Concentration		
Strains	*E*. *uniflora* extract (μg/mL)	Fluconazole (μg/mL)	FICI*	Effect
***Candida albicans* ATCC90028**	312.5	1.0	0.50	Synergistic (4x)
***Candida dubliniensis* CBS7987**	625	2.0	0.75	No interaction
***Candida tropicalis* ATCC13803**	1,250	4.0	0.50	Synergistic (4x)
***Candida parapsilosis* ATCC22019**	625	1.0	0.63	No interaction
***Candida glabrata* ATCC2001**	1,250	8.0	0.50	Synergistic (4x)

* Fractional inhibitory concentration index

**Table 4:** Synergistic activity of *E*. *uniflora* extract with Micafungin on *Candida* spp. MIC values *of E*. *uniflora* extract and Micafungin obtained in the test, calculation of the FICI and the effect resulting from the interaction of the substances. Values obtained using the checkerboard technique after incubation at 37°C for 48 h.

		Minimal Inhibitory Concentration		
Strains	*E*. *uniflora* Extract (μg/mL)	Micafungin (μg/mL)	FICI*	Effect
***Candida albicans* ATCC 90028**	312.5	0.25	0.50	Synergistic (4x)
***Candida dubliniensis* CBS 7987**	625	1.0	1.0	No interaction
***Candida tropicalis* ATCC 13803**	1,250	0.25	0.5	Synergistic (4x)
***Candida parapsilosis* ATCC 22019**	625	0.50	0.38	Synergistic (4x)
***Candida glabrata* ATCC 2001**	1,250	0.13	0.50	Synergistic (4x)

*Fractional inhibitory concentration index

**Table 5:** Synergistic activity of *E*. *uniflora* extract with Amphotericin B on *Candida* spp. MIC values of *E*. *uniflora* extract and Amphotericin B in the test, calculation of the FICI and the effect resulting from the interaction of the substances. Values obtained using the checkerboard technique after incubation at 37°C for 48 h.

		Minimal Inhibitory Concentration		
Strains	*E*. *uniflora* Extract (μg/mL)	Amphotericin B (μg/mL)	FICI*	Effect
***Candida albicans* ATCC 90028**	312.5	1.0	1.0	No interaction
***Candida dubliniensis* CBS 7987**	625.0	1.0	0.8	No interaction
***Candida tropicalis* ATCC 13803**	1,250	1.0	0.75	No interaction
***Candida parapsilosis* ATCC 22019**	625.0	0.5	0.76	No interaction
***Candida glabrata* ATCC 2001**	1,250	1.0	0.75	No interaction

*Fractional inhibitory concentration index

**Table 6:** Synergistic activity of *E*. *uniflora* extract with gallic acid on *Candida* spp. MIC values of *E*. *uniflora* extract and gallic acid obtained in the test, calculation of the FICI and the effect resulting from the interaction of the substances. Values obtained using the checkerboard technique after incubation at 37°C for 48 h.

		Minimal Inhibitory Concentration		
Strains	*E*. *uniflora* Extract (μg/mL)	Gallic Acid (μg/mL)	FICI*	Effect
***Candida albicans* ATCC 90028**	312.5	1,250	2.0	No interaction
***Candida dubliniensis* CBS 7987**	625	312.5	0.8	No interaction
***Candida tropicalis* ATCC 13803**	1,250	312.5	2.0	No interaction
***Candida parapsilosis* ATCC 22019**	625	156.25	3.0	No interaction
***Candida glabrata* ATCC 2001**	1,250	78.12	5.0	No interaction

*Fractional inhibitory concentration index

### Time-kill curve of *Candida* spp. cells growth in the presence of the *Eugenia uniflora* extract alone and combined with synthetic antifungal drugs

A time-kill curve was performed in order to understand how the EuE and/or antifungal drugs interfere with *Candida* spp cellular growth and viability at different time points, for the combinations of the EUE and synthetic antifungal drugs that showed synergism. Therefore, CFU counts of cells previously grown under different conditions and further incubation on YPD plates at 30°C, for 48 h were performed.

**Fig 6:**
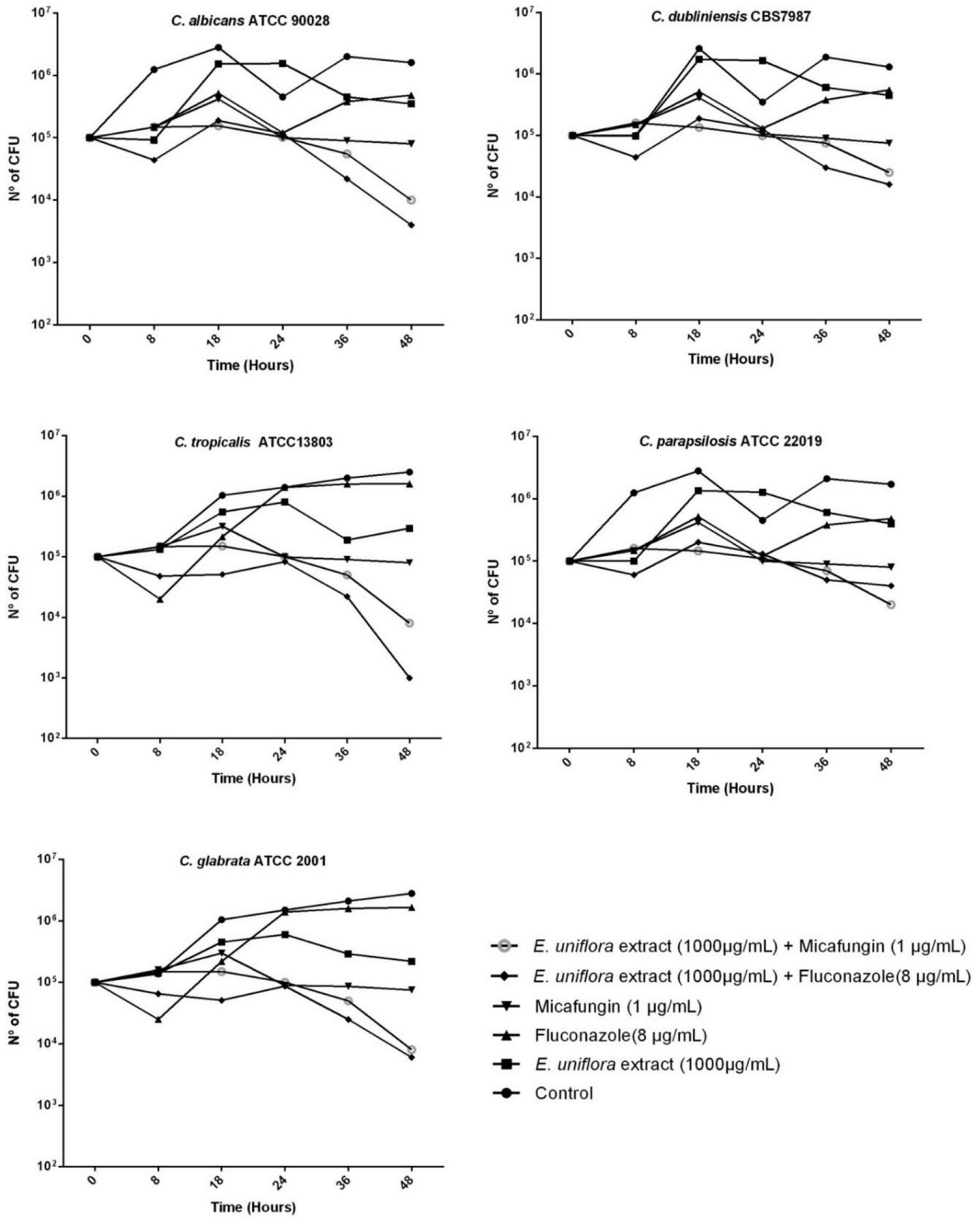
Action of the *Eugenia uniflora* extract on the time-kill curve of *Candida* spp. combined with antifungals. Results are represented as the mean ± standard deviation of CFU counts at each time point of analysis. Fungicidal activity was defined as a ≥ 3 log 10 reduction of the initial inoculum. Synergism and antagonism were defined as the decrease or increase of ≥ 2 log10 CFU/mL counts respectively, produced by the combined preparation, compared to that of the active agent alone.

It was possible to verify a similar time-kill curve when *Candida* spp. cells were treated with FLU only, when compared to the YPD broth control experiment. However, for the other conditions tested, a remarkable growth reduction was observed specifically after 36 h of incubation. The reduction of CFU counts was markedly seen for most of the strains and conditions tested, mainly when they were grown in the presence of a combination of the EuE with either MCF or FLU (Fig 6).

It is worth mentioning that the combined action between the EuE with either MCF or FLU was species-specific, where a reduction of at least 1 log10 of CFU counts was observed for *C*. *albicans* ATCC 90028 and *C*. *glabrata* ATCC 2001. For *C*. *tropicalis*, a 2 log10 reduction was observed, meaning a trend of synergistic action between the EuE and the antifungal compounds. Nevertheless, a less remarkable effect for *C*. *dubliniensis* CBS 7987 and *C*. *parapsilosis* growth inhibition was observed, when compared to the other *Candida* spp. strains tested.

## Discussion

The antifungal effect caused by plant-derived compounds is often related to qualitative and quantitative constituents [38]. The majoritary compounds isolated from the EuE are gallic acid and myricitrin [20, 39]. In this context, a few studies have demonstrated the anti-*Candida* action of these phytocompounds [40–43]. However, their interaction with some specific molecular targets that cause pharmacological effect on fungal cells is not fully understood. The chemical complexity of a plant extract is one of the reasons for the difficulty in elucidating a putative mechanism of action. The mixing of secondary metabolites mainly results in synergism. Therefore, more than one compound may be responsible for the biological effect studied. Furthermore, choosing a specific molecular target is considered an obstacle, since several secondary metabolites are known to have the ability to interact with different targets [24, 28].

During the *in silico* analysis, it was possible to detect that both gallic acid and myricitrin are compounds that directly interact with the active site of three well known target enzymes of antifungals. Gallic acid is a polyphenol compound widely present in several plant species [44]. Li et al. [45], analyzed the action of gallic acid *in vitro* and *in vivo* against *Trichophyton rubrum* and *C*. *albicans*. They have demonstrated that its action against *C*. *albicans* is comparable to that of FLU. The study also showed that gallic acid decreased *T*. *rubrum* 14-α-demethylase activity in a dose-dependent manner. Therefore, our *in silico* results reinforce acid gallic potential to inhibit ergosterol biosynthesis in different fungal species.

We have further focused on the investigation of a possible mechanism of action of the EuE on the fungal cell wall and plasma membrane. Firstly we have tested the hypothesis if the EuE could interact with plasma membrane, because the addition of an exogenous source of ergosterol in the culture medium where fungal cells have been growing can increase the MIC values if the analyzed compound targets this sterol present on fungal cells [30]. When exogenous ergosterol was added to the culture medium, *Candida* spp. cells showed less pronounced increased MICs when compared to AMB, making us to infer that the mechanism of action of the EuE is discreet on direct binding to plasma membrane. This result corroborates our *in silico* analysis findings, because there is a possibility that the probable interference of the extract on the fungal membrane is related to its interaction with the CYP51 enzymes (14-α-demethylase and Δ-14-sterol-reductase) in intermediate stages of ergosterol biosynthesis pathway rather than its direct binding to the plasma membrane.

There are no studies in the literature that have evaluated the action of *E*. *uniflora* extract direct binding to ergosterol, or even its interference with the biosynthesis of this molecule. However, Sardi et al. [46], analyzed the antifungal activity and possible mechanisms of action of plant extracts obtained from different parts of four different *Eugenia* spp. (*E*. *leinii*, *E*. *brasiliensis*, *E*. *myrcianthes* and *E*. *involucrate*). Despite the fact that the extracts impaired biofilm formation in *C*. *albicans*, they were not able to complex with exogenous ergosterol. These results partially corroborate with our findings.

When *Candida* spp. cells were grown in the presence of the EuE containing sorbitol, the MICS were increased for all the strains but *C*. *glabrata*. The increased MICs also happened in a similar trend with the control MCF, suggesting that one of the possible mechanisms of action of the extract is related to its interaction with targets present on the cell wall [30]. This finding corresponded to the interaction between myricitrin with the enzyme β-1,3 glucan synthetase found *in silico*. Sorbitol is known for having an osmoprotective function and it is essential for fungal growth when there is cell wall damage [47]. Therefore, this substance is used to identify fungal cell wall inhibitors. In fact, MIC values obtained in the presence of sorbitol are higher because protoplasts walls can continue to grow due to osmoprotection [48].

The fungal cell wall is a dynamic structure that protects fungal cells from changes in osmotic pressure and other environmental stressors, while allowing it to interact with the environment. Therefore, it may be an excellent target for the development of new antifungal drugs, specifically because it is not present in mammalian cells, reducing potential toxicity [49–51].

In the study conducted by Silva-Rocha et al. [15], it was demonstrated that the EuE clearly has altered cell wall morphology observed by microscopy and also impaired hypha formation. This finding is in agreement with our study, reinforcing the idea that it may act on the cell wall, since in the yeast-to-hyphae transition process, changes in cell morphology are directly linked to cell wall plasticity, composition and architecture [37, 39].

The EuE also clearly enhanced the effect of cell walls stressors. These results may be easily observed when cells were treated with either Calcofluor white or Congo red. However, cells treated with SDS (a cellular plasma membrane disrupting agent) remained mostly unaltered, corroborating the idea that the EuE mechanism of action might be more straightly driven to the cell wall. Changes in cell wall architecture usually happen after a stressful stimulus or exposure to antifungal drugs [8, 52].

Congo red plates (either alone or in combination with the EuE) showed a more strikingly reduction in CFU counts when compared to Calcofluor white. While both dyes stimulate chitin synthesis, Congo red binds to β-1,3-glucan and interferes with its assembly in the yeast *Saccharomyces cerevisiae* [53].

Our flow cytometer assay revealed that all the strains tested in the presence of the EuE emitted a percentage of fluorescence closer to cells treated with FLU rather than AMB. These results together with the other findings emphasize our hypothesis that the EuE probably acts on an enzyme important for ergosterol biosynthesis pathway (such as Δ-14-sterol-reductase, where possible interactions where found during our *in silico* analysis) instead of directly binding to ergosterol.

We investigated the combined action between the EuE extract with an azole derivative (FLU), an echinocandin (MCF), a polyene (AMB) and with gallic acid *in vitro*. It was observed that the combination of *E*. *unifora* extract with either FLU or MCF demonstrated synergistic action *in vitro* for most of the strains tested. It is important to note that in the time-kill curve test, the combination of the extract with both drugs also led to a decrease in the CFU counts after 36 h of incubation, notably for *C*. *albicans*, *C*. *tropicalis* and *C*. *glabrata*. Nevertheless, the results found led as to confirm the fungistatic rather than fungicidal nature of the EuE, even when combined to FLU and MCF.

## Conclusions

The compilation of the results found in our study allows us to infer that the extract of the leaves of *Eugenia uniflora* interacts with targets present in the cell wall and intermediates of biosynthesis of fungal membrane. However, this phenomenon apparently does not occur through direct binding to the ergosterol molecule. Instead, the interaction of this natural product with the enzymes that are part of the ergosterol and 1,3-β-glucan biosynthesis pathways may be the main mechanism of antifungal action. Therefore, the EuE may be a viable alternative therapy in the future when combined with FLU and MCF, for the prevention and treatment of *Candida* infections.

## Supporting information

S1 File Manuscript data set. (XLSX)
